# Peptide Nucleic Acids (PNAs) in Antimicrobial Therapy: A Next Generation Strategy

**DOI:** 10.3390/ijms27031565

**Published:** 2026-02-05

**Authors:** Antonia D’Aniello, Annalisa Masi, Concetta Avitabile, Giovanni del Monaco, Michele Saviano, Maria Moccia

**Affiliations:** 1 Institute of Crystallography, National Research Council of Italy, URT Caserta, Via Vivaldi 43, 81100 Caserta, Italy; antoniadaniello@cnr.it (A.D.); concetta.avitabile@cnr.it (C.A.); giovannidelmonaco@cnr.it (G.d.M.); michele.saviano@cnr.it (M.S.); 2 Institute of Crystallography, National Research Council of Italy, Strada Provinciale 35d, 9, Montelibretti, 00010 Rome, Italy; annalisa.masi@cnr.it

**Keywords:** Peptide Nucleic Acids (PNAs), Antimicrobial Resistance (AMR), antisense therapy, ribosome targeting, cell-penetrating peptides, nanoparticle delivery, multidrug-resistant bacteria

## Abstract

The global rise in antimicrobial resistance (AMR) demands innovative strategies beyond traditional antibiotics. Peptide Nucleic Acids (PNAs), synthetic DNA analogues with peptide-like backbones, act as thermically, chemically, and enzymatically stable sequence-specific agents capable of silencing essential bacterial genes. Through antisense mechanisms, PNAs bind bacterial mRNA or rRNA, blocking translation or ribosome assembly and thereby inducing species-specific growth inhibition. Their programmable design enables precise targeting of multidrug-resistant pathogens while sparing commensal microbiota. Recent advances, including γ-modified backbones, cationic substitutions, and delivery platforms such as cell-penetrating peptides (CPPs), dendron conjugates, and nanoparticles, have improved solubility, stability, and cellular uptake. Studies show promising in vitro and, albeit less frequently, in vivo efficacy against both Gram-positive and Gram-negative bacteria, often with synergistic activity when combined with conventional antibiotics. Although challenges remain in delivery and large-scale production, PNAs represent a promising class of antimicrobials to combat AMR through targeted gene inhibition.

## 1. Introduction

Excessive use of antibiotics has led to widespread bacterial resistance, creating a major global health threat [1]. Many bacteria have become multi-drug resistant (MDR), rendering existing antibiotics less effective and increasing the spread of resistant strains [2]. The ESKAPE pathogens (*Enterococcus faecium*, *Staphylococcus aureus*, *Klebsiella pneumoniae*, *Acinetobacter baumannii*, *Pseudomonas aeruginosa*, *Enterobacter* species) are particularly concerning, as they cause hospital-acquired infections and evade most antibiotics [3]. Prolonged development timelines and high costs limit the discovery of new antimicrobial agents, so the most effective antibiotics are often derived from modifications of previously known compounds [4]. Therefore, there is an urgent need to develop novel, potent antimicrobial agents with new mechanisms of action, such as antisense oligonucleotides, which inhibit essential bacterial protein production [5].

**Figure 1:**
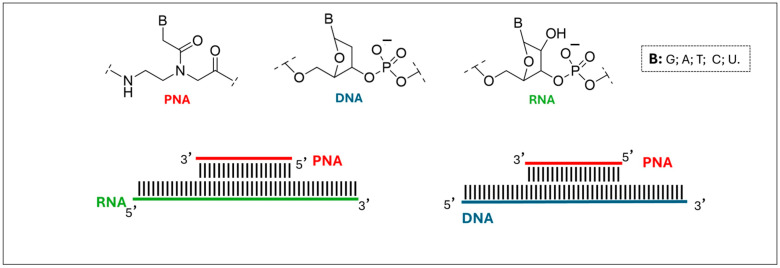
Antiparallel Watson–Crick duplex of PNA (red) with complementary single-stranded DNA (blue) or RNA (green).

One promising molecule is peptide nucleic acid (PNA), a synthetic molecule that combines the characteristics of both peptides and nucleic acids [6]. Designed by Nielsen through computer modelling, PNAs mimic DNA and RNA but possess a backbone made of N-(2-aminoethyl)-glycine linked by peptide bonds, with nucleobases (A, G, C, T) attached via methylene carbonyl linkers instead of sugars [6]. The *aeg* backbone is acyclic, achiral, and electrically neutral (Figure 1), eliminating electrostatic repulsion and resulting in highly stable PNA-DNA or PNA-RNA hybrids [6]. PNAs can bind complementary DNA or RNA sequences through Watson–Crick and Hoogsteen base pairing and even displace strands in double-stranded DNA (dsDNA) [7,8]. 

In this review, the term “antisense PNAs” refers to peptide nucleic acids designed to hybridize with bacterial mRNA or rRNA to inhibit gene expression. When specifically applied to bacterial pathogens, these molecules are referred to as “antibacterial PNAs”. For clarity, the term “antimicrobial” is used only in a general context, whereas the discussion throughout the manuscript focuses on antibacterial activity.

PNA molecules possess several advantages, including strong and highly stable binding affinity and specificity towards DNA or RNA, and higher thermal melting temperature (T_m_) than DNA-DNA or DNA-RNA duplexes [9,10]. They are stable across varying temperatures [11] and pH levels and show remarkable resistance to enzymatic degradation in serum and cell extracts [12], making them promising tools for both therapeutic and diagnostic applications [13].

PNAs have been mostly used for (a) antisense and antigene applications or to facilitate gene editing [14]; (b) diagnostics and biosensors [15]; (c) RNA targeting, by selectively binding to various RNA molecules, including miRNAs, siRNAs, and lncRNAs, thus enabling both diagnostics [16] and therapeutics [17,18] applications; and (d) the development of PNA-based therapeutics, such as OLP-1002 and NT-0200, which have demonstrated safety and efficacy in both clinical and preclinical studies [19]. In recent years, PNAs have exhibited antimicrobial and antibacterial properties, offering promise as alternatives or complements to traditional antibiotics [20]. In the context of the antibiotic resistance crisis and the rise in MDR pathogens, PNAs as antimicrobials have attracted growing interest as part of the search for novel, mechanism-based antimicrobial strategies [21].

Because PNAs can be designed to bind specifically to bacterial mRNA sequences, they enable species and potentially strain-specific targeting, which may help spare non-target members of the host microbiome compared to traditional broad-spectrum antibiotics [20]. Despite these advantages, PNAs face key challenges, including poor water solubility and inefficient cellular uptake due to their neutral backbone, which can cause aggregation and hinder membrane crossing [21] as well as high synthesis costs [22]. Conjugation with carrier molecules is therefore necessary to improve delivery and intracellular targeting [23,24]. Although the term antimicrobial activity broadly refers to inhibition of bacteria, fungi, and viruses, in this work, we specifically focus on the antibacterial properties of PNAs, summarizing the literature from 2021 onward [25,26].

Our aim is to discuss how PNAs can be designed to target bacterial genes and pathways. We examine how key factors such as PNA length, binding-site selection, target gene choice [21], and the method used to deliver PNAs into bacterial cells [24] influence their overall design and efficacy. 

## 2. Peptide Nucleic Acid Recognition of DNA and RNA

The properties of PNAs, mentioned above, enable them to interact with both DNA and RNA through multiple binding modes, including Watson–Crick duplexes, Hoogsteen triplexes, strand invasion, double-duplex invasion, and tail-clamp binding [11,14]. While all these mechanisms are mechanistically relevant, only a subset has been validated in antimicrobial applications (Table 1). 

### 2.1. Watson–Crick Duplex Binding to Single-Stranded RNA( ssRNA)

PNAs exert antimicrobial activity by forming stable duplexes with essential bacterial mRNAs, specifically blocking protein synthesis [27,28]. Short PNAs (9–11 nucleotides) target translation initiation regions and are effective without RNase H [29,30]. Delivery into bacterial cells remains challenging: carrier peptides like (KFF)_3_K or (RXR)_4_XB facilitate uptake, while newer strategies allow SbmA-independent entry [31,32,33]. Average MICs are ~5 µM for Gram-negative and ~14 µM for Gram-positive bacteria, highlighting their therapeutic potential and the importance of effective delivery [21]. Transcriptomic studies confirm high specificity with minimal off-target effects [33].

### 2.2. Watson–Crick Duplex Binding to Structured RNAs

In addition to mRNAs, PNAs have been directed against ribosomal RNAs (rRNA) and other structured bacterial RNAs [21]. Although these strategies are less common, they have demonstrated growth inhibition. However, the limited accessibility of structured RNA elements can reduce their efficacy [34].

### 2.3. Triplex Binding with dsRNA

Triplex-forming PNAs (TFPs) can target homopurine–homopyrimidine tracts in double-stranded RNA (dsRNA) through Hoogsteen interactions. These interactions are often stabilized by base analogues such as pseudocytosine or 2-aminopyridine. The resulting complexes show high affinity and selectivity for A-form RNA, and can modulate RNA conformations [35,36]. However, despite strong biophysical evidence, the in vivo antimicrobial applications of PNA triplexes have yet to be proven, primarily due to challenges in delivery and the intracellular inaccessibility of target dsRNAs [37].

Among the various binding modes, antisense Watson–Crick duplex formation with bacterial mRNA represents the most extensively validated mechanism of antimicrobial action for PNAs. However, PNA targeting of rRNA regions, such as functional domains within 16S or 23S rRNA, has also been shown to exert antibacterial effects, although these examples remain less systematically explored [25]. The antibacterial activity of PNAs depends on their capability to hybridize in a sequence-specific manner with bacterial messenger RNAs (mRNAs), thereby blocking translation and effectively preventing protein synthesis. These PNAs are generally designed to target the translation initiation region of essential genes, including the Shine-Dalgarno sequence and the start codon (AUG) [38]. By binding this region, they sterically hinder the assembly and progression of the 30S ribosomal subunit and can form a stable PNA–RNA heteroduplex that inhibits translation. However, the overall efficacy is highly dependent on the accessibility of the target site within the mRNA. In addition to directly blocking ribosome binding, antisense PNAs may modulate mRNA stability by altering local RNA structures and, in some cases, contribute to transcript degradation through ribonuclease-dependent mechanisms, although in bacteria, repression often occurs primarily via steric hindrance. Furthermore, heteroduplex formation induces conformational changes in the target RNA that further limit ribosomal access, thereby amplifying the translational blockade.

**Table 1:** PNA binding modes and evidence for antimicrobial applications.

Binding Mode	Mechanism of Interaction	Antimicrobial Evidence	References (APA)
Watson–Crick duplex (ssRNA/mRNA)	PNA hybridizes to complementary bacterial mRNA, blocking ribosome at start codon/RBS; RNase H–independent	Robustly validated.	[21,28,31,32,33,38,39]
Watson–Crick duplex (structured RNAs, e.g., rRNA)	PNA binds to accessible or partially accessible RNA regions (loops, bulges) and can invade short duplex or hairpin structures by strand displacement.	Some evidence. Growth inhibition reported, but accessibility of rRNA/structured RNA limits design space.	[34]
Triplex formation (dsRNA)	Hoogsteen hydrogen bonding to homopurine–homopyrimidine tracts; often requires base analogues (pseudocytosine, 2-aminopyridine)	Biophysically strong, but no antimicrobial in vivo evidence. High affinity for dsRNA, ability to modulate RNA conformation.	[35,36,37]

**Figure 2:**
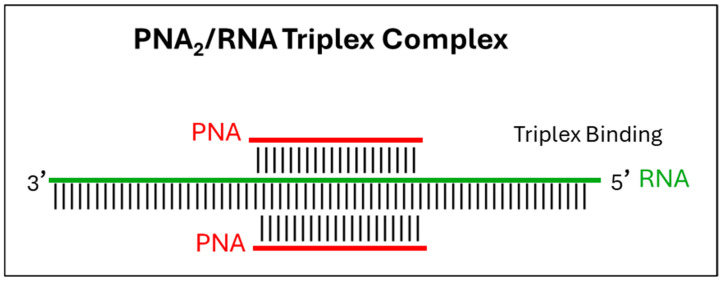
Alternative binding modes of PNAs to bacterial RNA.

Triplex binding remains mechanistically promising, but its antimicrobial potential is still largely theoretical and supported mainly by preclinical in vitro studies. Ongoing process in chemical modification and delivery technologies will be essential for translating these approaches into effective antibacterial applications (Figure 2).

### 2.4. Structural Insights and Backbone Modifications

Advances in structural biology (e.g., crystal and cryo-EM structures of PNA–nucleic acid complexes) and computational modelling (molecular dynamics and quantum mechanical studies) have elucidated the conformational determinants underlying PNA recognition. These findings have opened new opportunities for rational optimization of backbone geometry and base chemistry in next-generation PNA analogues [28]. Pre-organized PNAs, particularly γ-modified PNAs (γPNAs) bearing substituents such as miniPEG, exhibit enhanced solubility and binding affinity by biasing the backbone torsions into favourable conformations [39,40]. Computational work by Tamez et al. [41] further demonstrated that miniPEG-γPNA backbones adopt pre-structured conformations conducive to stable duplex formation. For these reasons, γPNAs have been widely employed as potential therapeutic agents. Shibata et al. [42] recently published the first crystal structure of a parallel duplex PNA/PNA invasion complex, providing novel mechanistic insight into the architecture and stability of PNA-based invasion. This structural milestone reinforces previous functional findings that parallel PNA orientations can facilitate efficient DNA invasion when guided by specific design constraints. Together, these studies highlight the critical role of chemical modifications and conformational pre-organization in enhancing PNA recognition of nucleic acids.

The body of work published between 2021 and 2025 highlights the versatility of PNAs as a molecular tool for nucleic acid recognition. Duplex hybridization with ssDNA/ssRNA is strengthened by their charge-neutral backbone and further enhanced by base modifications such as the G-clamp [43]. Additionally, triplex formation, particularly with dsRNA, expands the selectivity of PNA and enables structural modulation of RNA elements. Strand invasion into dsDNA is a hallmark of PNA technology, now significantly enhanced by chemical innovations that enable efficient recognition under physiological conditions and support application in genome editing. Double-duplex invasion further extends the sequence range accessible to PNA recognition. Tail-clamp PNAs (tc-PNAs) integrate triplex anchoring with duplex extension, allowing high-affinity targeting of mixed-sequence regions [37].

## 3. Targeted PNA Delivery to Bacterial Cells

Effective inhibition of bacterial genes by PNAs requires efficient cellular uptake; however, their neutral pseudopeptide backbone, while providing stability and resistance to degradation, limits solubility and prevents passive membrane diffusion. The limited availability of specialized bacterial transport systems further complicates delivery. While some transporters can facilitate the PNA uptake, their specificity and capacity are restricted, which hinders entry into bacterial cells. This approach faces similar challenges in carrying substances into mammalian cells without the use of delivery vectors.

As a result, strategies to improve bacterial uptake and bioavailability are essential. These include chemical modifications of the PNA backbone to enhance hydrophilicity, conjugation with positively charged amino acids, and coupling to carrier molecules capable of crossing bacterial membranes. Collectively, these approaches aim to overcome the natural impermeability of bacterial envelopes. By improving cellular entry, they enable PNAs to efficiently exert their antisense activity within the cytoplasm [24,30]. 

Various delivery strategies have been developed and validated in recent studies [44,45,46,47,48,49]. Among these, PNA–antimicrobial peptide (PNA-AMP) conjugates have demonstrated strong activity against Gram-negative pathogens. Patil et al. reported [44] that PNAs conjugated to the membrane-active AMPs (polymyxin) via Cys-CINA chemistry exhibited enhanced antibacterial efficacy against multidrug-resistant *Acinetobacter baumannii*. These conjugates are specifically designed to facilitate PNA penetration into bacterial cells, overcoming the permeability barrier of the Gram-negative outer membrane. The approach combined the antisense specificity and precision of PNAs targeting the acyl carrier protein gene (*acpP*) with the membrane-disrupting properties of AMPs. This strategy involved a reaction between an N-terminal cysteine residue and the nitrile group of cyanoisonicotinic acid, resulting in the formation of a stable thiazole ring [44]. The goal is to preserve the biological activity of both components, though some loss of activity may occur in some cases. Cys-CINA conjugation represents a promising approach for developing new antimicrobial therapies by effectively linking antisense oligonucleotides with AMPs [44]. Similarly, membrane-active peptide–PNA constructs incorporating peptides such as anoplin and (KFF)_3_K, when connected via non-cleavable Ethylene Glycol (EG1) linkers, demonstrated strong antibacterial activity against *E. coli* and *Salmonella Typhimurium*. In these constructs, cellular uptake was driven by the peptide moiety rather than by intrinsic PNA permeability, underscoring the critical role of both the carrier peptide and linker stability in achieving effective intracellular delivery. The EG1 linker, consisting of short ethylene glycol units, acts as a flexible, non-cleavable spacer that enhances the stability of the peptide–PNA conjugates and prevents premature dissociation. By maintaining an optimal spatial separation between the peptide carrier and the PNA sequence, the linker facilitates membrane penetration and preserves the antisense activity once the conjugate reaches the bacterial cytoplasm [45].

A novel method to directly monitor the intracellular uptake of PNAs in bacteria was recently developed by Sarkar et al. [46]. This system uses a synthetic RNA switch to regulate the expression of a fluorescent reporter, which is activated only if the antisense oligomer enters the bacterial cytoplasm and binds to its target sequence. Using this assay, the authors demonstrated that the inner membrane transporter SbmA plays a critical role in the uptake of peptide–PNA conjugates. In particular, the absence of SbmA led to a significant reduction in reporter activation, whereas wild-type strains continued to exhibit strong fluorescence signals. These results provide direct experimental evidence that SbmA functions as a key gateway for the intracellular delivery of PNAs in Gram-negative bacteria, highlighting its importance as a major factor influencing delivery efficiency.

In parallel, advances in nanotechnology have led to the development of multifunctional delivery platforms, including lipid and polymeric nanoparticles, carbon-based carriers, and supramolecular assemblies, which improve PNA stability, facilitate cellular uptake, and allow controlled intracellular release, offering a modular strategy for enhancing therapeutic performance [24].

A particularly promising approach for bacterial PNA delivery involves the use of mesoporous silica nanoparticles (MSNs), highly porous nanostructures with a large surface area, coated with extracellular vesicles (EVs) derived from *Staphylococcus aureus* [47]. This bio-mimetic nanoplatform not only enables efficient encapsulation and loading of an antisense PNA oligomer targeting essential *S. aureus* genes (encapsulation efficiency ≈ 62.9%, loading capacity ≈ 7.7%), but also improves stability and biocompatibility under physiological conditions. The EV shell provides selective bacterial recognition and promotes internalization, helping to overcome one of the major obstacles in delivering nucleic acid analogues to Gram-positive bacteria. Remarkably, this system demonstrated enhanced antimicrobial efficacy in vitro, showing a significantly greater inhibition of *S. aureus* growth at 8 µM compared to free PNA or non-coated MSN formulations. This highlights its promise as a potential “nano-antibiotic” strategy for precision antibacterial therapy. However, these results are still preliminary and require in vivo validation [47].

Complementing these strategies, chemical modifications of the PNA backbone have further advanced to the development of self-permeable PNAs. Incorporation of cationic units such as γ-modifications, guanidinium, or mini-PEG substituents enhances cellular uptake and binding affinity [48]. Building upon these strategies, recent years have witnessed the development of increasingly sophisticated and multifunctional delivery systems. In a recent study by Siekierska et al. [40], an antisense PNA complementary to the *acp*P mRNA, which encodes the acyl carrier protein in Gram-negative bacteria, was designed. The PNA was conjugated to two different cell-penetrating peptdes (CPPs), anoplin and (KFF)_3_K, using a non-cleavable ethylene glycol-based linker, 8-amino-2,6-dioxaoctanoyl (eg1). Among the conjugates, the “anoplin–eg1–PNA” construct exhibited the strongest antibacterial activity, with minimum inhibitory concentrations (MICs) of 2–4 µM against *Escherichia coli* and *Salmonella Typhimurium* [40].

**Figure 3:**
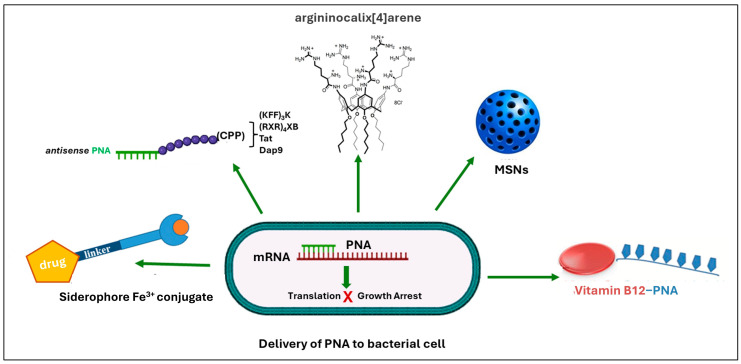
Overview of delivery strategies of PNAs in bacterial cells discussed in this review.

Alternative approaches include the use of non-peptidic ligands such as argininocalix[4]arene, which can mediate PNA transport without covalent conjugation [49]. These carriers rely on mechanisms such as the “proton sponge” effect to facilitate endosomal escape, though they generally achieve lower uptake compared with CPPs [49]. Importantly, argininocalix[4]arene-based vectors offer advantages in terms of biocompatibility and reduced cytotoxicity, as they do not require chemical modifications to the PNA. Additionally, their modular structure allows for the tuning of hydrophobicity and cationic charge to optimize cellular internalization. These features make them a promising next-generation delivery platform, potentially capable of overcoming some limitations of current PNA carriers. While these advantages are evident in mammalian models, their actual efficacy in bacterial systems remains less validated compared with CPP-based delivery systems [49]. Other studies have explored the conjugation of PNAs with natural molecules, such as vitamin B_12_ [50], to enhance uptake into Gram-negative bacteria like *Escherichia coli*. Vitamin B_12_ can function as a carrier by exploiting bacterial transport systems, including the BtuB-dependent pathway, improving intracellular delivery, and overcoming the permeability barriers of the outer membrane. This strategy enables PNAs to more efficiently reach their cytoplasmic targets, boosting their antisense activity and potential antimicrobial effects (Figure 3). Furthermore, the modular structure of vitamin B_12_ offers flexibility to improve PNA stability, solubility, and targeting precision [50].

In addition to vitamin B_12_ and peptide-based carriers, siderophore-mediated delivery has recently gained attention as a promising approach to promote PNA uptake in Gram-negative bacteria via native iron transport pathways. In their study, Tsylents et al. [51] developed antisense PNAs targeting the *acpP* gene. The 14-mer PNA sequence was functionalized at the N-terminus with an alkyne group and covalently linked to a synthetic trihydroxamate siderophore (SL) through “click” chemistry.

Biological evaluation showed that the SL–PNA conjugate-retained its iron-binding capacity and was internalized by *E. coli* cells in a FhuE/FhuD-dependent manner, as confirmed by flow cytometry and mutant analyses. However, despite efficient uptake, the SL–PNA targeting *acpP* of *E. coli* showed modest growth inhibition, with no significant reduction in optical density at 16 µM compared to controls. In a reporter assay, a rfp-targeting SL–PNA, fluorescence was reduced by approximately 40%, confirming successful delivery into the cells. These results demonstrate the potential of siderophore-mediated PNA transport while emphasizing the need for further optimization to enhance antibacterial activity [51].

## 4. PNAs as Antibacterial Agents

The antibacterial activity of PNAs is predominantly mediated through the antisense approach. PNAs specifically bind bacterial mRNA sequences via Watson–Crick base pairing, particularly at the ribosome binding site (RBS) or translation start region, physically blocking ribosomal assembly and translation, and thereby silencing essential genes involved in bacterial growth, replication, or virulence [52]. This mechanism enables sequence-specific inhibition of gene expression, distinguishing PNAs from broad-spectrum antibiotics and allowing precise targeting of pathogenic bacteria. This specificity is particularly valuable in the context of widespread antibiotic resistance, since antisense PNAs are thought to be less susceptible to common mechanisms such as enzymatic inactivation or target site mutations, although their activity can still be modulated by other bacterial defence strategies [52]. Additional challenges include nuclease degradation, reduced accessibility due to mRNA secondary structures, and the protective effect of biofilms, all of which can lower PNA efficacy. Unlike conventional antibiotics, which typically act on conserved structural components like the bacterial cell wall or ribosomal subunits, PNAs can be rationally designed to recognize nucleic acid sequences specific to a bacterial species or even a particular strain, enabling the development of narrow-spectrum or personalized antimicrobial therapies [21,23]. This high sequence specificity of PNAs allows them to selectively target and inhibit pathogenic microorganisms while leaving the beneficial commensal microbiota unharmed [53].

Given the clinical relevance of essential genes as antibacterial targets, this section highlights representative examples where antisense PNAs have been shown to effectively inhibit key genes required for bacterial growth and survival.

Numerous essential bacterial genes, including *acpP*, *gyrA*, *rpoD*, and *ftsZ*, are efficiently targeted and inhibited by PNAs in clinically relevant pathogens such as *Escherichia coli*, *Salmonella* spp., and *Pseudomonas aeruginosa* [53]. These findings demonstrate the versatility of PNAs as sequence-specific inhibitors capable of targeting genes essential for bacterial survival and growth [21,23].

Popella and colleagues [29] systematically evaluated the antibacterial activity of antisense PNAs targeting 11 essential genes in uropathogenic *Escherichia coli* (UPEC), including *acpP*, *dnaB*, *ftsZ*, and *rpsH*. Their results demonstrated that UPEC is highly susceptible to peptide-conjugated PNAs, with *acpP*-specific conjugates exhibiting minimum inhibitory concentrations (MICs) in the low micromolar range (5–10 µM). Interestingly, the abundance of the target transcript did not predict vulnerability, and growth inhibition by PNA was always associated with a measurable decrease in target mRNA levels.

Global transcriptomic analyses further revealed both sequence-dependent and sequence-independent responses, including the induction of membrane stress response pathways. The 9-mer PNAs were as effective as 10-mer counterparts in inhibiting bacterial growth, highlighting the potential of shorter PNAs in antimicrobial applications [29].

Furthermore, LASP-132, a dual-function PNA–peptide conjugate targeting *Acinetobacter baumannii*, exhibited strong antibacterial activity in Disc Diffusion Assays (DDA), generating inhibition zones of approximately 11–14 mm, although an exact MIC was not determined. Mechanistic studies showed that 11.9–16.5% of bacterial cells became propidium iodide–positive (PI-positive %) within one hour, indicating that LASP-132 exerts both antisense-mediated inhibition of *acpP* and direct membrane-disrupting effects [44]. Importantly, cytotoxicity toward human epithelial A549 cells remained below 10%, supporting the fact that it has preliminary therapeutic potential, although broader toxicity and in vivo evaluation are still required.

In another approach, Campion et al. [54] designed an antisense PNA–peptide conjugate targeting *dnaA*, the initiator of bacterial chromosome replication. Three variants targeted the translation start region exhibited MICs of 2 and 4 µM in *E. coli*, with the most potent variant, DnaA-3-PNA, inhibiting growth at approximately 2 µM. In contrast, a mismatch control required ≥16 µM to achieve comparable effects, highlighting the sequence specificity of the antisense mechanism. These results validate *dnaA* as a viable antisense target and demonstrate that replication-associated proteins can be efficiently silenced by PNAs.

More recently, Seo et al. [55] reported antisense PNA-CPP conjugates to target the essential *carA* gene in the multidrug-resistant pathogen *Acinetobacter baumannii.* Among the conjugates tested, KFFK(FFK)_2_–PNA demonstrated the strongest antibacterial effect, with a minimum bactericidal concentration (MBC) of 50 µM. At the same concentration, KKFK(FFK)_2_–PNA resulted in a colony count of 10.5 Colony-Forming Unit (CFU). However, chemical modifications such as the addition of lysine (Lys) residues were found to decrease efficacy. The 10-nucleotide α-PNA targeting the ribosome-binding site of *carA* showed the strongest inhibitory effect, significantly reducing both mRNA and protein levels, as confirmed by Quantitative Reverse Transcription Polymerase Chain Reaction (qRT-PCR) and Western blot analyses. Importantly, this inhibition was selective, showing no effect on standard Gram-positive or Gram-negative strains. Quantitative analyses further confirmed that PNA–CPP conjugates effectively suppress *carA* expression and limit bacterial proliferation. These results highlight the importance of rational CPP and PNA design in developing selective and potent antisense antibacterials against *A. baumannii* [55].

Further supporting the therapeutic potential of antisense PNAs in vivo models, Iubatti et al. [39] designed and synthesized diaminobutanoic acid (DAB) dendron–PNA conjugates that overcome the classical dependence on the inner-membrane transporter SbmA. When targeted against *acpP*, these conjugates exhibited submicromolar MICs. For example, (Goc)_8_-DAB-PNA 4850 inhibited wild-type *E. coli* at 0.25 µM, whereas a mismatch control required >16 µM. Strong activity was also observed against *Klebsiella pneumoniae* (MICs as low as 0.125 µM) and was retained in SbmA-deficient mutants, confirming an SbmA-independent uptake mechanism. The conjugates showed high serum stability (half-life > 24 h), efficacy in a murine peritonitis model caused by Extended-Spectrum Beta-Lactamase (ESBL)-producing *E. coli*, and good tolerability at intravenous doses up to 20 mg/kg. They also displayed favourable pharmacokinetics, characterized by renal clearance and minimal hepatic accumulation. Together, these findings identify DAB dendron–PNA conjugates as a highly promising next-generation antisense antibacterial scaffold [39].

**Figure 4:**
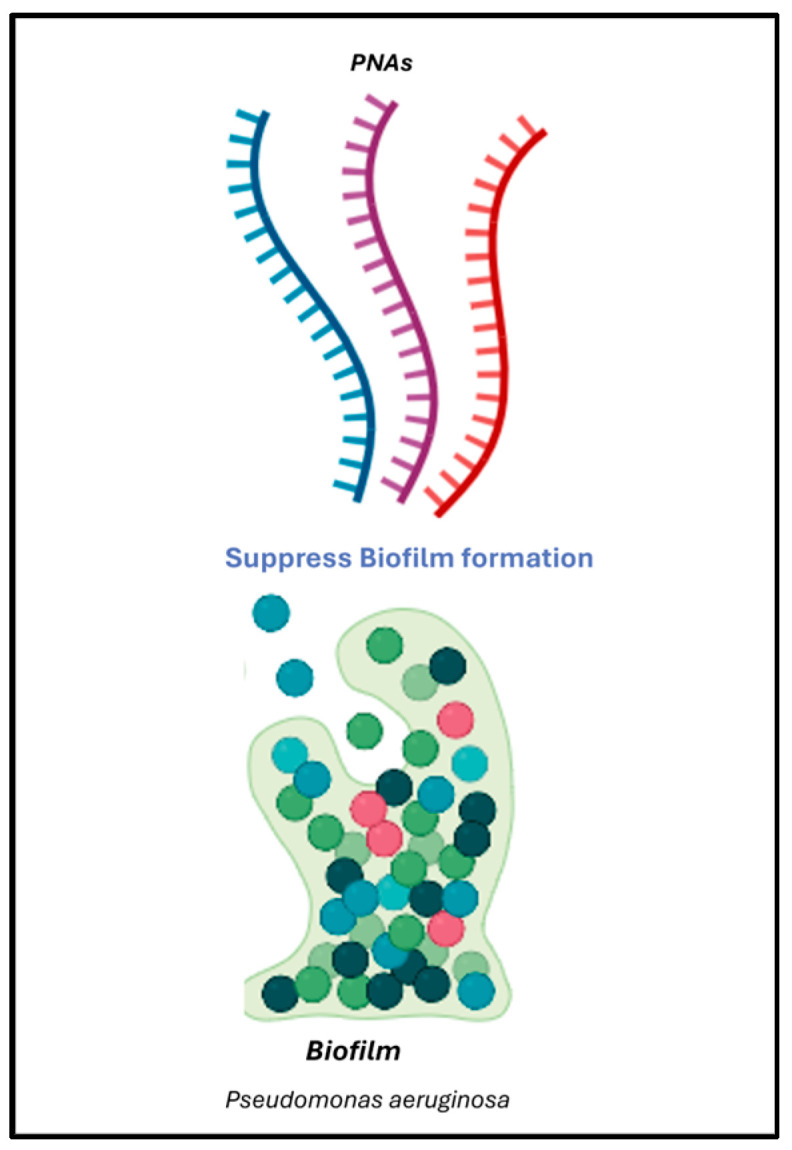
PNA cocktail targeting biofilm formation in *P. aeruginosa.*

PNAs have also shown strong potential to fight biofilm-associated infections (Figure 4). A PNA-based therapeutic cocktail was shown to effectively suppress *P. aeruginosa* biofilm formation in catheter-associated urinary tract infection (CAUTI) models, significantly reducing biofilm biomass and demonstrating strong activity against clinical isolates. In a study published by Karp and colleagues [56], broad-host-range PNAs were found to disrupt biofilm development not only in *P. aeruginosa* but also in other Gram-negative pathogens such as *Klebsiella pneumoniae* and *E. coli.* Quantitative assays showed a 50–70% reduction in biofilm biomass in vitro across multiple clinical strains.

In a murine CAUTI model, the PNA cocktail lowered bacterial loads on catheters by 1–2 log units compared to untreated controls. Importantly, its activity was largely confined to strains containing sequences complementary to the targeted PNAs, underscoring their high specificity [56].

Mechanistically, the PNAs interfere with regulatory pathways involved in biofilm formation, thereby reducing bacterial adhesion and impairing extracellular matrix synthesis. These findings indicate that PNA-based strategies may provide a valuable complement to conventional therapies for persistent, devices associated biofilm infections [57].

PNA-peptide conjugates have also shown activity against Gram-positive pathogens, such as *S. pneumoniae*. CPP-mediated uptake enhanced internalization and can reduce bacterial viability by as much as 3 log CFU/mL at 20 µM, without inducing cytotoxicity in mammalian cells. Different CPPs, including HIV-1 TAT and (RXR) 4XB, affect both translocation and antimicrobial potency, highlighting the importance of rational design of more effective and selective therapeutics [57].

Beyond their intrinsic antimicrobial activity, PNAs can act synergistically with conventional antibiotics [33]. In *E. coli*, targeting genes such as *acpP*, *fabI*, *folA*, or *folP* enhances the efficacy of ciprofloxacin and trimethoprim, allowing dose reduction and delaying the development of resistance. Similarly, PNA-mediated inhibition of *gyrA* increases the activity of levofloxacin against *Streptococcus pyogenes* [51].

In streptococcal pathogens, peptide-conjugated PNAs have been shown to effectively reduce bacterial viability and act synergistically with fluoroquinolones, highlighting their therapeutic promise as adjuvants to conventional antibiotics [56]. Notably, PNAs targeting efflux pump genes, such as *lfrA* in *Mycobacterium smegmatis* and *adeB* in Gram-negative bacteria, can restore antibiotic susceptibility, significantly decreasing bacterial density in both planktonic cultures and infected macrophages without inducing cytotoxic effects. These findings demonstrate that PNA-mediated efflux inhibition can effectively circumvent one of the major mechanisms of antibiotic resistance. As reported by Ghosh et al. [58], targeting efflux pumps with PNAs not only enhances the intracellular accumulation of antibiotics but also potentiates their bactericidal activity, highlighting PNAs as a promising adjunctive strategy to combat multidrug resistance in clinically relevant pathogens [58].

Mechanistic studies have revealed that PNAs employ sophisticated modes of action within bacterial systems. Notably, in vitro experiments have shown that PNAs can induce circularization of target mRNA molecules, thereby blocking ribosome initiation and effectively inhibiting translation, as demonstrated by De Chiara et al. [59]. In their experiments using bacterial mRNA sequences, the authors designed PNAs complementary to both the 5′ untranslated region and the 3′ end of the transcript, promoting the formation of a closed-loop structure that sterically hindered ribosome assembly. This induced circularization not only blocked translation initiation but also stabilized the PNA-mRNA complex, enhancing the overall efficiency of translational repression. Furthermore, PNAs targeting highly conserved regions of essential genes demonstrated strong inhibitory activity in vitro, in some cases at nanomolar concentrations, highlighting both the high specificity and therapeutic promise of this approach. However, higher doses are typically required for in vivo applications [59]. Taken together, the antibacterial activity of antisense PNAs reported in the literature spans a wide concentration range, from submicromolar to several tens of micromolar, depending on the bacterial species, target gene, and delivery strategy employed. The most potent systems, particularly peptide- or dendron-conjugated PNAs targeting essential genes such as *acpP* or *dnaA*, consistently achieve MIC values in the low micromolar or even submicromolar range. However, less optimized constructs often require concentrations of ≥10–50 µM to produce measurable antibacterial effects, which may limit their translational potential. Importantly, biological efficacy is largely governed by cellular uptake, conjugate design, and target accessibility rather than transcript abundance alone. While in vitro potency is often high, in vivo studies require higher doses, highlighting the need for further optimization to achieve an optimal balance between antimicrobial efficacy, selectivity, and pharmacokinetic properties.

## 5. Rational Design and Applications of Antisense PNAs Targeting Bacterial mRNAs

The exceptional specificity of antisense PNAs, whose mechanism of action has already been described above, is highlighted by the fact that even a single-nucleotide mismatch can dramatically reduce both binding affinity and inhibitory potency, emphasizing the importance of careful sequence design, target selection, and validation. High-throughput approaches using cell-free translation systems, such as INRI-seq (In vitro Ribosome Initiation Sequencing), have advanced the global assessment of translation initiation sites and off-target effects. These studies have shown that antisense PNAs can selectively inhibit the translation of the targets while minimizing unintended interactions. Key molecular factors for PNA efficacy, including target accessibility, RNA secondary structure, and thermodynamic stability, have been identified as critical for rational antisense design. Collectively, these insights underscore the potential of PNAs as highly programmable, sequence-specific inhibitors capable of precise modulation of bacterial gene expression. While promising, current findings are primarily limited to in vitro and animal models, and further research is needed to validate their therapeutic potential in clinical settings [60].

Other successfully targeted genes include those involved in cell division (*ftsZ*), cell wall biosynthesis *(murA*, *fabI*), transcription regulation (*rpoD*, *gyrA*), and antibiotic resistance determinants (*bla*, *ermB*, *mecA*). These targets include both essential housekeeping functions and genes contributing to drug resistance, making them strategically attractive for therapeutic intervention. Beyond *E. coli*, antisense PNAs have demonstrated potent activity against essential genes in other clinically relevant pathogens. In *P. aeruginosa*, PNAs targeting *acpP* and *ftsZ* efficiently inhibited bacterial growth and biofilm formation, indicating their potential to combat Gram-negative infections. Similarly, in *Klebsiella pneumoniae*, antisense PNAs directed at *acpP*, *rpoD*, *murA*, and *adk* significantly reduced bacterial proliferation both in vitro and in animal infection models. In *Salmonella enterica*, PNAs targeting *acpP* effectively decreased mRNA levels and suppressed bacterial growth [33]. Notably, recent work by Col et al. [61] extended these findings to *S. aureus*, where antisense PNAs conjugated with CPPs were designed to target critical biofilm-associated genes (*ica*, *sarA*, *rot*, *yycF/G*, among others). Interestingly, only the PNA directed against *sarA* produced consistent inhibition of biofilm formation (up to 40% at 50 µM), highlighting its potential as an adjuvant therapy in biofilm-associated infections [61].

A key aspect in the development of antisense PNAs is the rational design of sequences that maximize on-target efficacy while minimizing off-target interactions. Recent progress in computational tools has significantly advanced this process. Notably, Jung et al. [62] introduced the Multispectral Antisense Oligonucleotide Off-target Prediction (MASON) web server, a specialized platform for designing antisense oligomers targeting bacterial mRNAs with integrated off-target prediction. MASON enhances sequence specificity by assessing potential cross-hybridization with non-target transcripts, thereby reducing unintended bacterial stress responses and improving therapeutic safety. Additionally, MASON enables users to evaluate multiple parameters simultaneously, such as target site accessibility, thermodynamic stability, and potential secondary structure interactions, factors essential for efficient PNA binding. The platform also supports high-throughput screening of large genomic datasets, streamlining the identification of optimal antisense candidates across diverse bacterial species. The availability of such advanced bioinformatic tools represents a critical step forward in antisense PNA design, offering a systematic, predictive alternative to traditional trial-and-error methods and accelerating progress toward clinical application [62].

This targeted mechanism may reduce the risk of rapid resistance development, though mutations at the target sites remain possible. Moreover, their high sequence specificity helps minimize off-target impacts on the beneficial microbiota.

Furthermore, PNAs can be rapidly reprogrammed against new resistance determinants. Recent advances, such as the high-throughput tiling approach described by Danti et al. [63], have further improved the efficacy of PNA-based antisense strategies by systematically identifying optimal binding sites across essential bacterial mRNAs, thereby significantly enhancing inhibitory potency. This strategy demonstrated that not all accessible regions of mRNAs are equally effective as antisense targets, and that comprehensive coverage can reveal highly potent sites that would remain unpredictable. Importantly, the study also highlighted the scalability of this method, enabling the rapid discovery and validation of antisense targets across different bacterial species. By combining systematic target identification with the inherent programmability of PNAs, this approach paves the way for the rational design of antisense antibiotics on a versatile platform, adaptable to multiple pathogens [63].

## 6. Ribosome as a Target for Antibacterial PNA

The bacterial ribosome is a fundamental macromolecule responsible for decoding genetic information and synthesizing proteins. Owing to its essential role and high structural conservation, the ribosome has long been a preferred target for conventional antibiotics, including macrolides, aminoglycosides, and oxazolidinones [64,65]. More recently, antisense PNAs have emerged as an innovative class of agents capable of specifically interfering with ribosomal function. Their unique design enables PNAs to hybridize strongly with rRNA or with translation initiation regions of essential messenger RNAs, thereby disrupting ribosome activity and inhibiting bacterial growth [21,33]. Mechanistically, when PNAs bind to structured or functionally critical regions of rRNA, they can sterically hinder ribosomal subunit assembly, block tRNA binding, or interfere with the elongation cycle.

**Figure 5:**
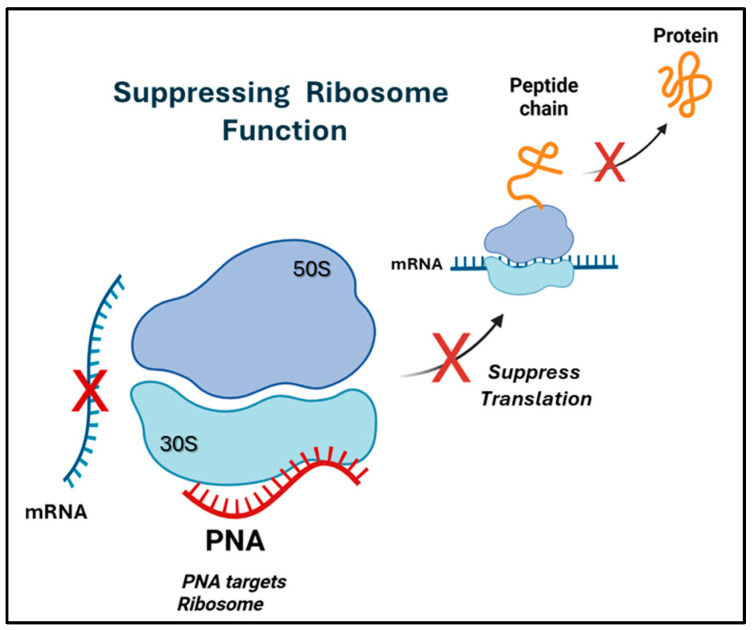
Ribosome as a molecular target of PNAs.

PNAs targeting mRNA regions involved in ribosome recruitment can effectively suppress translation initiation, adding to their versatility as tools for modulating ribosomal function (Figure 5) [29,33]. The molecular properties of PNAs are critical in determining their effectiveness in ribosomal inhibition. Short oligomers (~8–12 nucleotides) that exhibit minimal self-structure and are conjugated to suitable carrier molecules have consistently shown the highest performance [21,32].

Several ribosomal domains have been validated as especially promising PNA targets. Among these, the 23S rRNA stands out due to its multiple functional hotspots. The peptidyl transferase center (PTC), which is responsible for catalyzing peptide bond formation, and the α-sarcin/ricin loop, critical for elongation and recruitment of Guanosine Triphosphatase (GTPase) factors, are both highly conserved across bacterial species [64,65].

In addition to these well-characterized regions, other ribosomal structures have also been investigated as potential PNA targets. One such element is Helix 68 (H68) of the 23S rRNA, which plays a role in forming intersubunit bridges and regulating elongation dynamics [25,66]. Importantly, the nearby ribosomal interface bridge (H69/B2a interface) has been identified as a binding site for aminoglycosides, highlighting the pharmacological significance of this region [30].

Recent structural studies emphasize that careful selection of conserved and accessible rRNA regions is critical for designing highly specific PNA sequences with clinical potential [67]. Beyond rRNA, antisense PNAs commonly target translation initiation regions of essential bacterial genes, effectively blocking ribosome binding at Shine–Dalgarno sequences and start codons.

For example, PNA–bacteria-penetrating peptide (BPP/CPP) conjugates targeting genes such as *acpP*, *ftsZ*, and rne in carbapenem-resistant *A. baumannii* have demonstrated complete growth inhibition with MICs of 2–8 µM across clinical isolates and 2–4 µM in a recent follow-up study[68].

In *E. coli*, optimized anti-*acpP* PNA equipped with an arginine-rich CPP achieved an MIC of 2.5 µM [29]. Meta-analyses suggest that effective PNA designs typically yield MICs around 4–5 µM across Gram-negative species, with higher values observed for suboptimal targets or less efficient delivery strategies [21]. Together, these findings emphasize the critical roles of target accessibility, sequence selection, and delivery chemistry in determining antibacterial efficacy.

## 7. Barriers to the Clinical Application of Antibacterial PNAs

Despite their promise, the clinical translation of PNA-based antimicrobial therapies remains significantly hampered by high production costs and safety concerns. The synthesis of PNAs, especially backbone-modified variants like γ-PNAs, is technically demanding and expensive, relying on chiral monomers, elaborate protection and deprotection steps, and meticulous purification processes. These requirements make large-scale manufacturing difficult and limit their feasibility as widespread therapeutics.

In addition, producing PNAs at high yield and purity requires highly optimized solid-phase synthesis protocols and sophisticated chromatographic purification, further driving up manufacturing costs. Together, these economic and technical hurdles remain major obstacles to advancing PNAs toward clinical use as antimicrobial agents [69].

From a pharmacokinetic perspective, the in vivo biodistribution and fate of PNAs remain poorly characterized. Reliable data on absorption, half-life, tissue accumulation, and clearance are largely lacking, and many constructs exhibit rapid systemic elimination combined with limited retention in target tissues, which complicates the maintenance of therapeutic concentrations. Furthermore, only a few preclinical models have thoroughly evaluated these parameters under physiologically relevant conditions [27].

Beyond issues of cost and pharmacokinetics, the immunogenicity and cytotoxicity of PNAs and their delivery systems must be carefully evaluated. In one investigation, a CPP-PNA conjugate targeting the *cagA* gene of *Helicobacter pylori* was administered to mice, and no significant increases in serum ImmunoglobulinG (IgG) or IgM (ImmunoglobulinM) levels were observed, even at high doses. Levels of liver enzyme AST (Aspartate Aminotransferase) and ALT (Alanine Aminotransferase) remained comparable to those of control animals, suggesting minimal immune activation and negligible hepatic toxicity [70].

PNAs present potential cytotoxicity related to intracellular accumulation and the chosen delivery method. In human cell lines, many PNA–peptide conjugates have been found to be trapped in the endosomal compartment, which can limit their efficacy and potentially induce intracellular stress [30,48]. Furthermore, while certain antisense PNA–AMP conjugates demonstrated strong antibacterial activity, their therapeutic use must be carefully managed: doses that are too high may compromise membrane integrity or interfere with normal cellular metabolism.

These challenges [21] become even more significant when highly charged or extended peptide carriers are employed, as they can destabilize cellular membranes and activate stress-response pathways. Consequently, optimizing both the PNA sequence and the carrier design is crucial to reducing cytotoxicity in mammalian systems [71].

To overcome these limitations, ongoing research is focusing on several complementary approaches: improving synthesis and purification methods to boost scalability and reduce production costs; developing delivery vectors with high biocompatibility and minimal toxicity; and designing more advanced pharmacokinetic/pharmacodynamic (PK/PD) models to better predict the in vivo behaviour of PNAs.

For example, Avitabile et al. highlight the application of advanced nanotechnology-based platforms, including nanoparticles, liposomes, and calixarenes, to enhance cellular uptake while maintaining an optimal balance of stability, controlled release, and tolerability. Together, these integrated strategies are designed to support the safe and effective clinical translation of this emerging antimicrobial technology [24].

## 8. Conclusions

The continuous rise in antimicrobial resistance demands innovative strategies able to overcome the limitations of traditional antibiotics [1,5]. In this context, PNAs are emerging as a programmable antisense platform with high chemical stability and resistance to enzymatic degradation [12,20]. By hybridizing with specific mRNA or rRNA sequences, PNAs enable selective inhibition of essential genes or virulence factors while minimizing microbiota disruption and reducing off-target effects [21,33]. To better define their role among emerging antibacterials, it is useful to compare them with antibacterial peptides, which kill rapidly through membrane disruption and penetrate biofilms efficiently [72].

Although engineered peptides such as SAAP-148 show strong activity against Gram-negative pathogens even under complex conditions [72], their broad mechanism may cause limited selectivity, cytotoxicity at higher doses, and disruption of commensal Gram-negative microbiota. In contrast, antibacterial PNAs rely on sequence-directed inhibition of intracellular pathways [21,29,53], offering a precision-based approach that reduces collateral damage and can be rapidly reprogrammed to target emerging or strain-specific threats. In addition, PNAs, antibacterial peptides, and traditional antibiotics differ across key pharmacological parameters. Antibacterial peptides act within minutes through membrane disruption but may induce resistance via membrane remodelling and present cytotoxicity risks [72]. Traditional antibiotics show diverse spectra and mechanisms but face widespread resistance [1,5].

PNAs act more slowly due to the need for intracellular delivery, yet their sequence-defined targeting minimizes off-target effects and reduces the likelihood of resistance, as mutations in essential genes impose high fitness costs [22]. These key distinctions are summarized in Table 2, which provides a direct comparison between antibacterial PNAs, antibacterial peptides, and traditional antibiotics across the main pharmacological parameters discussed.

These distinctions highlight PNAs as precision antibacterials with a favourable selectivity–resistance profile. Recent advances have also improved PNA delivery. CPP conjugates [39], DAB dendrimers bypassing SbmA [40], biomimetic nanoparticles coated with extracellular vesicles [20], synthetic siderophores [50], and non-peptidic vectors such as arginino-calix[4]arenes [49] have enhanced uptake, stability, and intracellular release. Backbone innovations, including miniPEG γ-modifications [27,28,41], have produced analogues with improved affinity, solubility, and potential self-permeability.

Despite the significant progress described in recent years, the biological activity of antibacterial PNAs is still largely observed at micromolar concentrations, which remain higher than those typically required for conventional antibiotics. Furthermore, challenges such as synthesis costs, biodistribution issues, and the lack of robust preclinical models [21,22,24] continue to limit progress. Nevertheless, the inherent advantages of PNAs, including the high specificity, modular design, rapid redesign capability, and the ability to access targets beyond classical pharmacology, position PNAs as highly promising candidates for next-generation antibacterials.

## Figures and Tables

**Table 2:** Comparison of antibacterial PNAs, antibacterial peptides, and traditional antibiotics.

Parameter	Antibacterial PNAs	ABPs	Traditional Antibiotics
Spectrum	Narrow; species/strain-specific [21,29,50]	Broad-spectrum [55]	Variable [1,5]
Mechanism	Antisense genetic inhibition [21,29]	Membrane disruption [72]	Diverse (cell wall, DNA, protein) [1,5]
Speed	Slow (requires uptake)	Very rapid	Variable
Resistance	Low; high fitness cost [22,29]	Moderate	High
Cytotoxicity	Very low	Possible at high doses	Variable
Delivery	Requires carriers	None	None
Reprogramming	High	Low	None

## Data Availability

Not applicable.

## Abbreviations

The following abbreviations are used in this manuscript:

AMP	Antimicrobial Peptide
AMR	Antimicrobial Resistance
ALT	Alanine Aminotransferase
APA	American Psychological Association
AST	Aspartate Aminotransferase
B2a	Ribosomal interface bridge (H69/B2a)
BPP	Bacteria-Penetrating Peptide
CAUTI	Catheter-Associated Urinary Tract Infection
CFU	Colony-Forming Unit
CPP	Cell-Penetrating Peptide
DAB	Diaminobutanoic Acid
DNA	Deoxyribonucleic Acid
dsDNA	Double-Stranded DNA
dsRNA	Double-Stranded RNA
EG1	Ethylene Glycol (linker)
ESBL	Extended-Spectrum Beta-Lactamase
ESKAPE	*Enterococcus faecium*, *Staphylococcus aureus*, *Klebsiella pneumoniae*, *Acinetobacter baumannii*, *Pseudomonas aeruginosa*, and *Enterobacter* species
EV	Extracellular Vesicle
GTPase	Guanosine Triphosphatase
H68/H69	Helix 68/Helix 69 (regions of 23S rRNA)
INRI-seq	In vitro Ribosome Initiation Sequencing (cell-free translation assay)
IgG	ImmunoglobulinG
IgM	ImmunoglobulinM
MASON	Multispectral Antisense Oligonucleotide Off-target Prediction (web server)
MBC	Minimum Bactericidal Concentration
MDR	Multi-Drug Resistance
MIC	Minimum Inhibitory Concentration
MSN	Mesoporous Silica Nanoparticle
PD	Pharmacodynamic
PEG/miniPEG	Polyethylene Glycol/mini Polyethylene Glycol
PI-positive	Propidium Iodide–Positive (marker of membrane disruption)
PK	Pharmacokinetic
PNA	Peptide Nucleic Acid
PTC	Peptidyl Transferase Center
qRT-PCR	Quantitative Reverse Transcription Polymerase Chain Reaction
RBS	Ribosome Binding Site
rRNA	Ribosomal RNA
RNA	Ribonucleic Acid
ssRNA	Single-Stranded RNA
tcPNA	Tail-Clamp Peptide Nucleic Acid
TFP	Triplex-Forming PNA
UPEC	Uropathogenic *Escherichia coli*
γPNA	γ-Modified Peptide Nucleic Acid.
